# Comparative analysis of a presbyopia-correcting intraocular lens that combines extended depth-of-focus and bifocal profiles with a standard monofocal intraocular lens

**DOI:** 10.1186/s12886-022-02516-6

**Published:** 2022-07-14

**Authors:** Da Eun Shin, Hun Lee, Kyungmin Koh

**Affiliations:** 1 Department of Ophthalmology, Kim’s Eye Hospital, Konyang University College of Medicine, 136 Youngshinro, Youngdeungpo-gu, Seoul, 07301 Republic of Korea; 2 Department of Ophthalmology, Asan Medical Center, University of Ulsan College of Medicine, Seoul, Republic of Korea

**Keywords:** Continuous range of vision, Extended depth of focus, Intermediate vision, Intraocular lens, Near vision

## Abstract

**Background:**

Recently, a new presbyopia-correcting intraocular lens (IOL) that combines extended depth-of-focus and bifocal profiles (ZFR00: Tecnis® Synergy®, Johnson & Johnson Vision, Santa Ana, CA, USA) has been established and several studies have been reported. We attempted to compare the performance with a standard IOL (ZCB00: Tecnis® monofocal, Johnson & Johnson Vision, Santa Ana, CA, USA) manufactured using the same material from the same company, which has been extensively used worldwide.

**Methods:**

The medical records of patients undergoing cataract surgery with ZCB00 or ZFR00 implantation between March 2021 and September 2021 and with available 3-month visit data were reviewed. Uncorrected near, intermediate, and distance visual acuity (VA), corrected distance VA, and optical quality were the main outcome measures.

**Results:**

This study included forty-six patients (64 eyes), with twenty-one patients (32 eyes) implanted with ZCB00 and twenty-five patients (32 eyes) implanted with ZFR00. The average age of the patients was 66.0 ± 10.1 (range: 40 to 82) and 65.1 ± 4.7 (range: 59 to 77) years in the ZCB00 and ZFR00 groups, respectively. The preoperative characteristics did not differ significantly between the two groups. Compared to the ZCB00 group, the ZFR00 group demonstrated significantly superior intermediate and near VA (*p* < 0.001) at 3 months postoperatively. The ZFR00 group showed significantly lower objective measured optical quality than that in the ZCB00 group.

**Conclusions:**

The ZFR00 exhibited a continuous range of vision and a smooth defocus curve, while the ZCB00 provided superior objective optical quality.

**Supplementary Information:**

The online version contains supplementary material available at 10.1186/s12886-022-02516-6.

## Introduction

Cataracts are a major cause of vision loss and cataract surgery is the most common ocular surgery worldwide [1]. Monofocal intraocular lenses (IOLs) remains the most prevalent IOL due to their relatively low cost, excellent vision at a selected distance, and rare probabilities of photic phenomena [2]. Since the monofocal IOLs provide only one focus, either long-distance glasses or reading glasses are needed, depending on the target refraction. With the increase in life expectancy and the participation of older adults in professional life, presbyopia has become a very common visually impaired condition [3]. Consistent with this trend, the ratio of multifocal (bifocal or trifocal), or extended depth-of-focus (EDOF) IOL implantations is increasing [4]. Different bifocal IOL platforms were implanted in the past, but currently, the most common presbyopia correcting IOLs implanted, include on the one hand the trifocal models, like the PanOptix (Alcon Laboratories Inc., Fort Worth, TX, USA), FineVision Micro F IOL (PhysIOL, Liège, Belgium), Acriva Reviol Tri-ED 611 (VSY Biotechnology, Amsterdam, The Netherlands), and AT Lisa tri (Carl Zeiss Meditec AG, Jena, Germany), and on the other hand, modern EDOF like the Symfony (Johnson & Johnson Vision Inc., Santa Ana, CA, USA), or Vivity (Alcon Laboratories Inc., Fort Worth, TX, USA) IOLs [4, 5].

Recently, an IOL combining the two main approaches of presbyopia-correcting IOLs, multifocal and EDOF, has also been released. The newly introduced IOL, Tecnis® Synergy® (ZFR00: Johnson & Johnson Vision, Santa Ana, CA, USA) received Conformité Européenne mark approval in 2019 and U.S. Food and Drug Administration approval in 2021 [6]. Combining both bifocal and EDOF diffractive technologies, it offers continuous vision over a range that includes all distances: from near to far [7].

Since the release of ZFR00, clinical outcomes have been reported with this IOL alone [6–8], or in comparison with other presbyopia-correcting IOLs [9, 10]. However, no studies have compared the ZFR00 to a standard monofocal IOL (ZCB00: Johnson & Johnson Vision, Santa Ana, CA, USA) of the same material and basic design, which is the most widely implanted worldwide [11, 12]. This study compared the clinical results of both IOLs.

## Materials and methods

### Subjects

This single-center, retrospective, comparative study was undertaken in accordance with the tenets of the Declaration of Helsinki and was approved by the Institutional Review Board (IRB file number: 2021-12-001) of Kim’s Eye Hospital, Seoul, Republic of Korea, which waived the requirement for written informed consent because of the retrospective design and the use of de-identified patient data. Moreover, this study contained no personal information that could lead to the identification of any patient and the data were analyzed anonymously. We reviewed the medical records of patients who had undergone cataract surgery with ZCB00 or ZFR00 implantation between March and September 2021 at Kim’s Eye Hospital, Seoul, Republic of Korea.

Patients aged 40 years or older with visual significant cataract and corneal astigmatism of less than 1 diopter (D) were included in the study. and eligible for three or more months of follow-up. Patients had a relevant ophthalmic condition that could influence their results, a history of eye surgery or trauma, and the follow-up period was less than 3 months after surgery were excluded.

The manifest refraction (MR) was measured with the Early Treatment Diabetic Retinopathy Study (ETDRS) chart in photopic light conditions. Uncorrected distance visual acuity (UDVA) and corrected distance visual acuity (CDVA) were assessed at 6 m. Uncorrected intermediate visual acuity (UIVA) was measured at 66 cm. Uncorrected near visual acuity (UNVA) was assessed at 40 and 33 cm. All post-surgical visual acuity tests were performed monocularly at 3 months following surgery. Monocular defocus curves derived at the same visit.

For statistical purposes, the evaluated decimal values were transferred to the logarithm of the minimum angle of resolution (LogMAR) scale. The power calculations for the inserted IOL and the expected postoperative refractive error (RE) were identified according to the Barrett Universal II formula by means of an anterior segment swept-source optical coherence tomography device (ANTERION, Heidelberg Engineering GmbH, Germany). The assumed IOL power was the closest to emmetropia.

To compare the refractive predictability, the RE and mean absolute error (MAE) were observed. The RE was described as the gap between the postoperative spherical equivalent (SE) and the presupposed SE. The MAE was identified as the mean absolute value of the RE [13].

The defocus curves were obtained monocularly at 3 months after surgery using defocusing lenses with a power range of 1.50 D to − 4.00 D in 0.5 D steps. These lenses were inserted into a test frame to account for the manifest error in the refraction of the distance. The measurement was carried out with ETDRS charts at 6 m in mesopic light conditions [14, 15].

### Objective optical quality assessment

The HD Analyzer (Visiometrics SL., Terrassa, Spain) measurements were carried out in the mesopic state to assess objective optical quality parameters. All measurements were taken with a 4-mm aperture [16]. The objective scatter index (OSI), modulation transfer function (MTF), and Strehl ratio (SR) were calculated [16]. The OSI quantifies intraocular scatter, and the lower OSI values indicate better optical quality [2]. The MTF is the ratio of the contrast of the image to the object in terms of the frequency of an object [17]. .The SR indicates a perfect optical system at a value of 1 [18]. Hence, higher MTF and SR values usually indicate better objective optical quality [19, 20].

### Surgical procedures

The same experienced surgeon (KK) carried out whole surgeries. Main corneal incision was carried out using a 2.8 mm blade through the steep meridian. An anterior capsular opening of 5.2 mm was performed with continuous curvilinear capsulorrhexis. Following phacoemulsification (Whitestar Signature™ phocoemulsification system, Johnson & Johnson Vision, CA, USA), the IOL was implanted into the capsular bag. Then, every corneal wound was sealed with stromal hydration.

### Intraocular lenses

The ZCB00 is a foldable one-piece acrylic IOL with a translucent, continuous 360-degree posterior square edge and 6.0-mm optics [21]. It has an aspheric, modified prolate anterior surface that is designed to minimize spherical aberrations and improve contrast sensitivity after cataract surgery [22]. In contrast, the ZFR00 is a bifocal IOL combined with EDOF technology for continuous vision. Its posterior surface is diffractive with fifteen rings [23]. The distinct added power of the ZFR00 IOL is kept as proprietary information, and the photic phenomena are reportedly reduced through the use of Optiblue® material that passes blue light and blocks violet light [8]. Both IOLs are made from the same material (hydrophobic acrylic material, refractive index = 1.47 at 35 °C) with the same basic design [24].

### Statistical analysis

IBM SPSS Statistics for Windows, version 22.0 (IBM Corp., Armonk, New York, USA) was used to perform the statistical analysis. Kolmogorov–Smirnov tests were used to check the normality of the data distributions. Mann-Whitney U, unpaired Student t-, and Pearson chi-square tests were used to verify the differences between the two groups. For all cases, *p* < 0.05 was considered statistically significant. Data are presented as means ± standard deviation (SD).

## Results

This study consisted of 64 eyes from 46 patients, of which 21 (32 eyes) were implanted with ZCB00 and 25 (32 eyes) were implanted with ZFR00. There were no statistically significant differences in the preoperative characteristics of the patients in the groups (Table 1). The mean age of patients was 66.0 ± 10.1 (range: 40 to 82) years and 65.1 ± 4.7 (range: 59 to 77) years for the ZCB00 and ZFR00 groups, respectively. In the ZCB00 group, 67% of the patients (14/21) were female, whereas in the ZFR00 group, 44% of the patients (11/25) were female. In the ZCB00 group, 47% (15/32) of implants were in the right eye while in the ZFR00 group, 50% (16/32) of implants were in the right eye. In the ZCB00 group, the mean preoperative monocular UDVA (LogMAR), CDVA (LogMAR), and MRSE (diopter) were 0.34 ± 0.15, 0.21 ± 0.10, and 0.43 ± 1.43, respectively. In the ZFR00 group, these values were 0.29 ± 0.13, 0.18 ± 0.12, and 0.69 ± 1.48, respectively.

**Table 1:** Preoperative characteristics in each group

Parameter	ZCB00	ZFR00	***p***-value
Patients/Eyes, n	21/32	25/32	N/A
Female, n	14 (67%)	11 (44%)	.616^*^
Right eye, n	15 (47%)	16 (50%)	.803^*^
Age (years)	66.0 ± 10.1 (40 to 82)	65.1 ± 4.7 (59 to 77)	.141^†^
UDVA (LogMAR)	0.34 ± 0.15 (0.1 to 0.7)	0.29 ± 0.13 (0.1 to 0.6)	.077^†^
CDVA (LogMAR)	0.21 ± 0.10 (0.05 to 0.4)	0.18 ± 0.12 (0 to 0.4)	.311^†^
MRSE (D)	0.43 ± 1.43 (−3.0 to 3.5)	0.69 ± 1.48 (−2.0 to 3.0)	.594^‡^

*N/A* not applicable, *UDVA* uncorrected distance visual acuity, *CDVA* corrected distance visual acuity, *LogMAR* logarithm of the minimum angle of resolution, *SD* standard deviation, *MRSE* manifest refraction spherical equivalent, *D* diopter

^*^Chi-square test

^†^Mann-Whitney U test

^‡^Unpaired student t-test

**Fig. 1:**
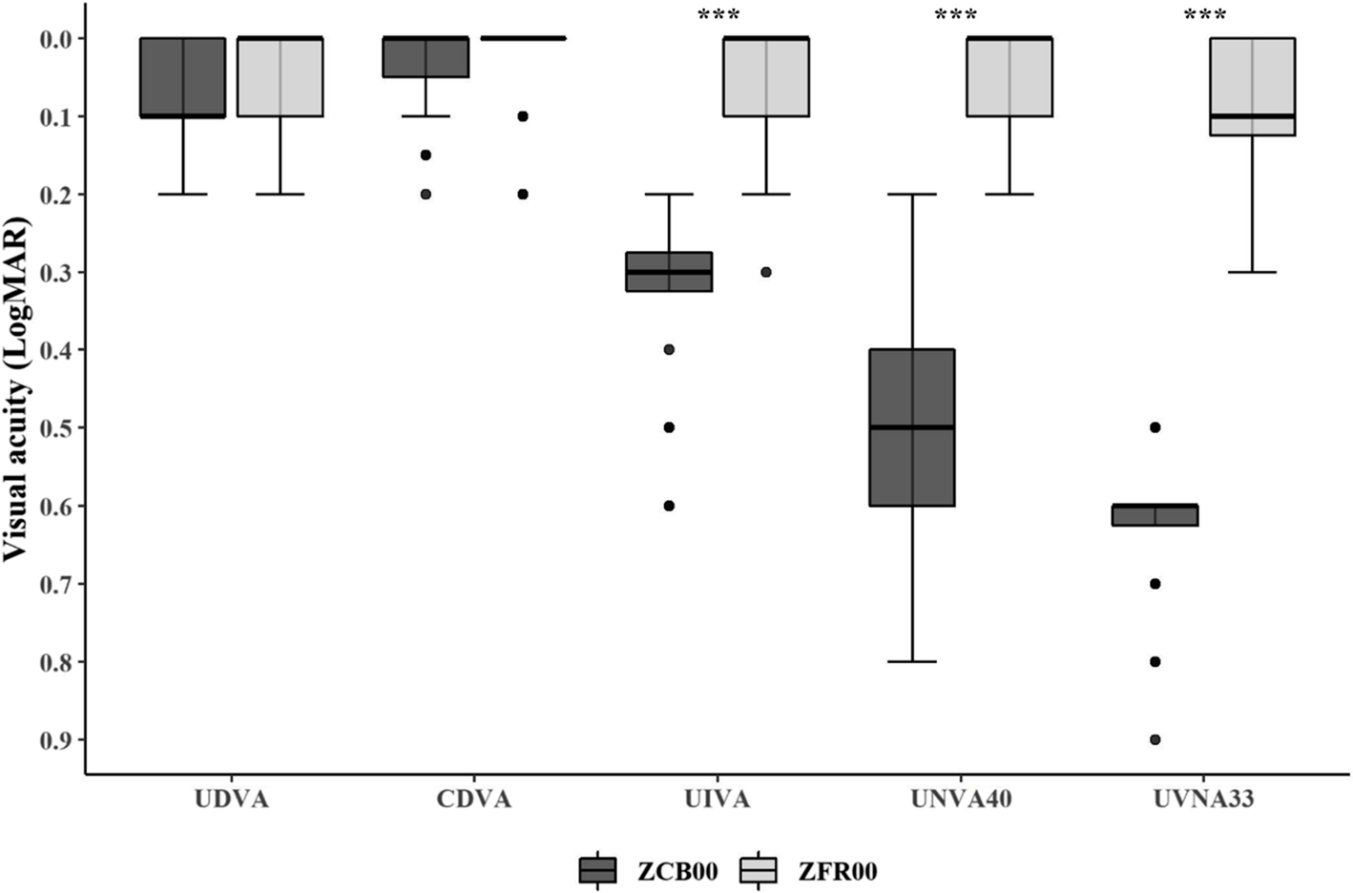
Comparison of monocular visual acuity between Tecnis® monofocal (ZCB00) intraocular lens (IOL) and Tecnis® Synergy® (ZFR00) IOL groups. LogMAR = logarithm of the minimal angle of resolution; UDVA = uncorrected distance visual acuity; CDVA = corrected distance visual acuity; UIVA = uncorrected intermediate visual acuity; UNVA40 = uncorrected near visual acuity at 40 cm; UNVA33 = uncorrected near visual acuity at 33 cm; *** = *p* < .001

The 3-month postoperative visual evaluation results for both groups are presented in Table 2. The monocular UDVA showed excellent results in both groups (ZCB00: 0.08 ± 0.06, ZFR00: 0.06 ± 0.07), without significant differences between the groups. The monocular CDVA was also excellent in both groups (ZCB00: 0.03 ± 0.05, ZFR00: 0.03 ± 0.05), with no statistical differences between groups. Compared to the ZCB00 group, the ZFR00 group showed a significantly better monocular UNVA (*p* < 0.001). In the ZCB00 group, the postoperative average monocular UIVA (at 66 cm, LogMAR), UNVA (at 40 cm, LogMAR), and UNVA (at 33 cm, LogMAR) were 0.33 ± 0.12, 0.48 ± 0.15, and 0.62 ± 0.09, respectively. In the ZFR00 group, these values were 0.02 ± 0.05, 0.03 ± 0.06, and 0.09 ± 0.10, respectively. All three parameters were significantly greater in the ZFR00 group than in the ZCB00 group (Table 2 and Fig.1). The target SEs were 0.06 ± 0.12 D in the ZCB00 group and 0.09 ± 0.14 D in the ZFR00 group. The MRSE was − 0.21 ± 0.34 D in the ZCB00 group and − 0.09 ± 0.31 D in the ZFR00 group. The mean error was − 0.29 ± 0.34 D in the ZCB00 group and 0.17 ± 0.30 D in the ZFR00 group. The mean absolute error was 0.35 ± 0.28 D in the ZCB00 group and 0.28 ± 0.20 D in the ZFR00 group. Post-surgical REs did not differ significantly between the two groups.

**Table 2:** Postoperative 3 months visual outcomes

Parameter	ZCB00	ZFR00	***p***-value
UDVA (LogMAR)	0.08 ± 0.06 (0 to 0.2)	0.06 ± 0.07 (0 to 0.2)	.204^*^
CDVA (LogMAR)	0.03 ± 0.05 (0 to 0.2)	0.03 ± 0.05 (0 to 0.2)	.145^*^
UIVA (LogMAR) 66 cm	0.33 ± 0.12 (0.2 to 0.6)	0.02 ± 0.05 (0 to 0.2)	<.001^*^
UNVA (LogMAR) 40 cm	0.48 ± 0.15 (0.2 to 0.8)	0.03 ± 0.06 (0 to 0.2)	<.001^*^
UNVA (LogMAR) 33 cm	0.62 ± 0.09 (0.5 to 0.9)	0.09 ± 0.10 (0 to 0.3)	<.001^*^
Target SE (D)	0.06 ± 0.12 (− 0.20 to 0.20)	0.09 ± 0.14 (− 0.15 to 0.38)	.262^*^
MRSE (D)	− 0.21 ± 0.34 (− 1.00 to 0.50)	−0.09 ± 0.31 (− 0.75 to 0.38)	.271^*^
Refractive error (D)	−0.29 ± 0.34 (− 1.20 to 0.60)	0.17 ± 0.30 (− 0.71 to 0.29)	.401^†^
Mean absolute error (D)	0.35 ± 0.28 (0.03 to 1.20)	0.28 ± 0.20 (0 to 0.71)	.317^*^

*UDVA* uncorrected distance visual acuity, *CDVA* corrected distance visual acuity, *UIVA* uncorrected intermediate visual acuity, *UNVA* uncorrected near visual acuity, *LogMAR* logarithm of the minimum angle of resolution, *SD* standard deviation, *SE* spherical equivalent, *MRSE* manifest refraction spherical equivalent, *D* diopter, *RE* refractory error, *n* number

^*^Mann-Whitney U test

^†^Unpaired student t-test

**Fig. 2:**
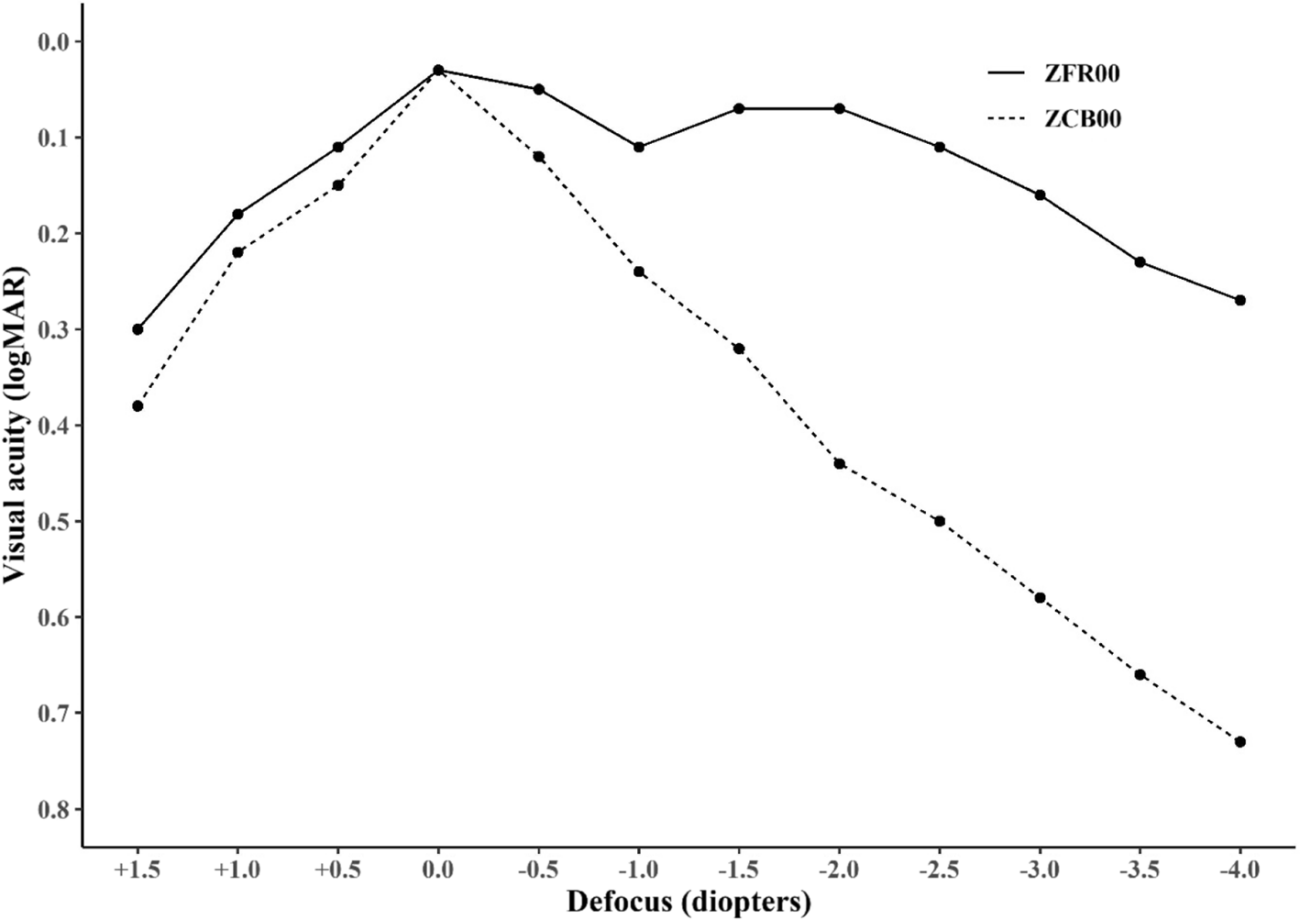
Mean monocular defocus curves obtained from the Tecnis® monofocal (ZCB00) intraocular lens (IOL) and Tecnis® Synergy® (ZFR00) IOL groups. logMAR = logarithm of the minimal angle of resolution; D = diopters

The defocus curves were assessed every 0.5 D at 3 months postoperatively at 6 m in mesopic light conditions (Fig. 2). The defocus curves showed that ZFR00 provided a wider DOF range than the ZCB00. The defocus curve of the ZFR00 indicated that the average VA stayed better than or equal to 0.11 LogMAR in the + 0.5 D to − 2.5 D interval. Moreover, between + 1.5 D and − 4.0 D VA, which is the whole section, the value was always better than or equal to 0.30 LogMAR. The defocus curve of the ZCB00 showed a mean VA equal or better than 0.24 LogMAR within the + 1.0 D to − 1.0 D interval and equal or better than 0.44 LogMAR within the + 1.5 D and − 2.0 D interval. There were no statistical differences between the two groups at + 1.0D, + 0.5D, and 0.0D. However, the ZFR00 was significantly better than the ZCB00 in the other sections. (*p* < 0.001).

The objective optical quality parameters assessed by HD Analyzer on a 4.0 mm pupil are shown in Table 3. The OSI, MTF cutoff, and SR of the ZCB00 were 1.22 ± 0.42, 27.37 ± 4.73, and 0.18 ± 0.03, respectively, and 5.19 ± 2.18, 10.40 ± 4.71, and 0.08 ± 0.02, respectively, for the ZFR00. The values of the three optical quality parameters were lower in the ZFR00 than those for the ZCB00 (*p* < 0.001).

**Table 3:** Objective optical quality parameters assessed by a HD Analyzer with a pupil diameter of 4.0 mm after 3 months of surgery

Parameter	ZCB00	ZFR00	***p***-value
OSI	1.22 ± 0.42 (0.76 to 2.20)	5.19 ± 2.18 (2.00 to 10.20)	<.001^*^
MTF cutoff (c/deg)	27.37 ± 4.73 (17.22 to 33.15)	10.40 ± 4.71 (4.00 to 19.81)	<.001^*^
Strehl ratio	0.18 ± 0.03 (0.13 to 0.24)	0.08 ± 0.02 (0.05 to 0.12)	<.001^*^

*OSI* objective scatter index, *MTF* modulation transfer function, *SD* standard deviation

^*^Mann-Whitney U test

## Discussion

Multifocal IOLs have two, three, or four optical intensities which aim at obtaining a decent VA at selected distances [25]. Because such features lead to reduced contrast sensitivity, the frequency of glare is higher for multifocal IOLs than for monofocal IOLs [26, 27]. EDOF IOLs, on the other hand, aim to extend the range of distant VA to intermediate distance and enable enhanced continuous VA [28]. A newly introduced ZFR00 has been designed to blend the diffractive factors of multifocal IOL (Tecnis® Multifocal, Johnson & Johnson Vision, Santa Ana, CA, USA) and EDOF (Tecnis Symfony®, Johnson & Johnson Vision, Santa Ana, CA, USA) IOL [6]. The manufacturers say that the hybrid optical technology in ZFR00 aims to combine the good distant and near visual acuity scores of a diffractive multifocal IOL with a continuous vision from far to near vision performance of an EDOF IOL [9].

As of December 2021, five papers on ZFR00 have been published. Three articles reported only the clinical findings of ZFR00 and one study comparing the IOL to Acrysof IQ® Panoptix IOL (Alcon Laboratories, Inc., Fort Worth, Texas, USA) and FineVision® POD F IOL (PhysIOL, Liège, Belgium) [7–9]. The other is a comparison of the outcomes of six types of presbyopia-correcting IOLs [10].

Both groups offered excellent distance vision. A previous clinical study reported results consistent with those from this study. Earlier studies reported monocular UDVAs of 0.07 ± 0.09 [21] and 0.10 ± 0.14 [29] for the ZCB00 and 0.04 ± 0.10 [9] and − 0.01 ± 0.04 [8] for the ZFR00. Earlier studies reported monocular CDVA of − 0.02 ± 0.09 [21] and 0.03 ± 0.06 [29] for the ZCB00 and − 0.02 ± 0.07  and − 0.04 ± 0.02 [10] for the ZFR00. Likewise, within our study, the UDVA values were 0.08 ± 0.06 and 0.06 ± 0.07 and the CDVA values were 0.03 ± 0.05 and 0.03 ± 0.05 in the ZCB00 and ZFR00 groups, respectively.

Previous studies have reported monocular UIVAs (at 66 cm) of 0.25 ± 0.18 [30], 0.34 ± 0.12 [29] for the ZCB00 and 0.05 ± 0.03 [8], 0.04 ± 0.09 [6] for the ZFR00, monocular UNVA (at 40 cm) of 0.48 ± 0.32 [31], 0.51 ± 0.19 [29] for the ZCB00 and 0.03 ± 0.05 [8], 0.05 ± 0.13 [6] for the ZFR00. In the current study, the monocular UIVA (at 66 cm) values were 0.33 ± 0.12 for the ZCB00 and 0.02 ± 0.05 for the ZFR00 and the monocular UNVA (at 40 cm) values were 0.48 ± 0.15 for the ZCB00 and 0.03 ± 0.06 for the ZFR00. The UNVA and UNVA were markedly better in the ZFR00 group than those in the ZCB00 group (*p* < .001).

The defocus curve is a good means of assessing the DOF of the presbyopia-correcting IOL as a visual achievement indicator [32–34]. The ZFR00 provided a smooth defocus curve with a broader landing area than the ZCB00 (Fig. 2). In this study, ZFR00 maintained VA better than or equal to 0.11 LogMAR in the + 0.5 D to − 2.5 D interval. However, ZCB00 kept VA above or equal to 0.15 LogMAR only in the short interval of + 0.5D to − 0.5D (Fig. 2). Throughout the section, the ZFR00 was significantly superior to the ZCB00 in all sections except + 1.0D, + 0.5D, and 0.0D (*p* < 0.001). The defocus curve of the ZFR00 in our study was like that of a study described a mean VA above 0.10 LogMAR between + 0.50 D and − 3.00 D [6]. Another study also revealed a mean flat curve of 0.00 to 0.10 LogMAR [7].

The HD Analyzer is useful for to assess the objective optical quality of IOLs and has good repeatability [35]. The objective OSI value for the multifocal IOL was strongly associated with subjective levels of glare [16]. The ZCB00 exceeded the ZFR00 in all three measures obtained from the HD Analyzer. The ZCB00 also offered consistent and excellent visual performance, with minimal degrees of visual disturbance compared to other multifocal IOLs [36].

This study has a number of limitations which affect the interpretation of the findings. The first of these is retrospective design and non-randomization. Second, due to the characteristics of the diffractive IOL in which the light is partially lost, accurate evaluation of objective optical quality has some restrictions. Since the HD analyzer recognizes the diffusion that occurs in the diffraction rings, higher OSI values can be measured in ZFR00 with diffraction rings. Third, subjective assessment of the quality of vision (contrast sensitivity test) has not been conducted. The study of additional visual parameters, including contrast sensitivity, photic phenomena, and an internal higher-order aberrations would allow a better understanding of the properties of each IOL. Fourth, This study has adopted only a monocular approach. Binocular measurements reflect a more real view due to the presence of indications of retinal disparity in addition to blurring. Fifth, a small number of subjects were included and the follow-up period lasted only 3 months after surgery. Our findings may provide pilot data for future research and a long-term prospective survey with a larger sample size in binocular approach is needed to identify true differences between two IOLs.

In summary, the ZFR00 exhibited a continuous range of vision and a smooth defocus curve, while the ZCB00 provided superior objective optical quality.

## Supplementary Information

**Additional file 1.**

## Data Availability

The datasets generated and analyzed during the current study are not publicly available due to protection of the patient’s personal information but are available from the corresponding author on reasonable request.

## Abbreviations

CDVA: Corrected distance visual acuity
EDOF: Extended depth-of-focus
IOL: Intraocular lens
MAE: Mean absolute error
MTF: Modulation transfer function
UDVA: Uncorrected distance visual acuity
UNVA: Uncorrected near visual acuity
OSI: Objective scatter index
PSF: Point spread function
RE: Refractive error
SE: Spherical equivalent
SR: Strehl ratio

## References

[1] Chung JK, Lee HK, Kim MK, Kim HK, Kim SW, Kim EC, Kim HS (2019). Cataract surgery practices in the Republic of Korea: a survey of the Korean Society of Cataract and Refractive Surgery 2018. Korean J Ophthalmol.

[2] Mencucci R, Cennamo M, Venturi D, Vignapiano R, Favuzza E (2020). Visual outcome, optical quality, and patient satisfaction with a new monofocal IOL, enhanced for intermediate vision: preliminary results. J Cataract Refract Surg.

[3] Greenstein S, Pineda R (2017). The quest for spectacle Independence: a comparison of multifocal intraocular Lens implants and Pseudophakic Monovision for patients with presbyopia. Semin Ophthalmol.

[4] Singh B, Sharma S, Dadia S, Bharti N, Bharti S (2020). Comparative evaluation of visual outcomes after bilateral implantation of a diffractive trifocal intraocular Lens and an extended depth of focus intraocular Lens. Eye Contact Lens.

[5] Kim S, Yi R, Chung S. Comparative analysis of the clinical outcomes of mix-and-match implantation of an extended depth-of-focus and a diffractive bifocal intraocular lens. Eye Contact Lens. 2022;48(6):261-6. 10.1097/ICL.0000000000000887 35333819

[6] Ribeiro FJ, Ferreira TB, Silva D, Matos AC, Gaspar S (2021). Visual outcomes and patient satisfaction after implantation of a presbyopia-correcting intraocular lens that combines extended depth-of-focus and multifocal profiles. J Cataract Refract Surg.

[7] Gabrić N, Gabrić I, Gabrić K, Biščević A, Piñero DP, Bohač M (2021). Clinical outcomes with a new continuous range of vision presbyopia-correcting intraocular lens. J Refract Surg.

[8] Ozturkmen C, Kesim C, Karadeniz PG, Sahin A. Visual acuity, defocus curve and patient satisfaction of a new hybrid EDOF-multifocal diffractive intraocular lens. Eur J Ophthalmol. Online ahead of print. 10.1177/11206721211057338 34766507

[9] Ferreira TB, Ribeiro FJ, Silva D, Matos AC, Gaspar S, Almeida S (2022). Comparison of refractive and visual outcomes of three presbyopia-correcting intraocular lenses. J Cataract Refract Surg..

[10] Palomino-Bautista C, Sánchez-Jean R, Carmona-Gonzalez D, Piñero DP, Molina-Martín A (2021). Depth of field measures in pseudophakic eyes implanted with different type of presbyopia-correcting IOLS. Sci Rep.

[11] Tanabe H, Tabuchi H, Shojo T, Yamauchi T, Takase K (2020). Comparison of visual performance between monofocal and multifocal intraocular lenses of the same material and basic design. Sci Rep.

[12] Denoyer A, Le Lez ML, Majzoub S, Pisella PJ (2007). Quality of vision after cataract surgery after Tecnis Z9000 intraocular lens implantation: effect of contrast sensitivity and wavefront aberration improvements on the quality of daily vision. J Cataract Refract Surg.

[13] Kim BH, Hyon JY, Kim MK (2019). Effects of bifocal versus trifocal diffractive intraocular Lens implantation on visual quality after cataract surgery. Korean J Ophthalmol.

[14] Galvis V, Tello A, Carreño NI, Berrospi RD, Niño CA, Serna VH (2020). Defocus curve and vergence related to viewing distance. J Cataract Refract Surg.

[15] Galvis V, Escaf LC, Escaf LJ, Tello A, Rodríguez LD, Lapid-Gortzak R (2022). Visual and satisfaction results with implantation of the trifocal Panoptix® intraocular lens in cataract surgery. J Opt..

[16] Tan QQ, Lin J, Tian J, Liao X, Lan CJ (2019). Objective optical quality in eyes with customized selection of aspheric intraocular lens implantation. BMC Ophthalmol.

[17] Alarcon A, Canovas C, Rosen R, Weeber H, Tsai L, Hileman K, Piers P (2016). Preclinical metrics to predict through-focus visual acuity for pseudophakic patients. Biomed Opt Express.

[18] Tabernero J, Garcia-Porta N, Artal P, Pardhan S (2021). Intraocular scattering, blinking rate, and tear film Osmolarity after exposure to environmental stress. Transl Vision Sci Technol.

[19] Saad A, Saab M, Gatinel D (2010). Repeatability of measurements with a double-pass system. J Cataract Refract Surg.

[20] Liao X, Li JY, Tan QQ, Tian J, Lin J, Lan CJ (2020). Comparison of visual quality after implantation of A1-UV and SN60WF aspheric intraocular lens. Int J Ophthalmol.

[21] Huh J, Eom Y, Yang SK, Choi Y, Kim HM, Song JS (2021). A comparison of clinical outcomes and optical performance between monofocal and new monofocal with enhanced intermediate function intraocular lenses: a case-control study. BMC Ophthalmol.

[22] Yamauchi T, Tabuchi H, Takase K, Ohsugi H, Ohara Z, Kiuchi Y (2013). Comparison of visual performance of multifocal intraocular lenses with same material monofocal intraocular lenses. PLoS One.

[23] Kanclerz P, Toto F, Grzybowski A, Alio JL (2020). Extended depth-of-field intraocular lenses: an update. Asia Pac J Ophthalmol.

[24] Łabuz G, Son HS, Naujokaitis T, Yildirim TM, Khoramnia R, Auffarth GU (2021). Laboratory investigation of preclinical visual-quality metrics and halo-size in enhanced Monofocal intraocular lenses. Ophthalmol Therapy.

[25] Vingolo EM, Carnevale C, Fragiotta S, Rigoni E, Iacobelli L (2017). Visual outcomes and contrast sensitivity after bilateral implantation of multifocal intraocular lenses with +2.50 or +3.0 diopter addition: 12-month follow-up. Semin Ophthalmol.

[26] Kretz FT, Khoramnia R, Attia MS, Koss MJ, Linz K, Auffarth GU (2016). Clinical evaluation of functional vision of +1.5 diopters near addition, aspheric, rotational asymmetric multifocal intraocular Lens. Korean J Ophthalmol.

[27] Ukai Y, Okemoto H, Seki Y, Nakatsugawa Y, Kawasaki A, Shibata T, Mito T, Kubo E, Sasaki H (2021). Quantitative assessment of photic phenomena in the presbyopia-correcting intraocular lens. PLoS One.

[28] Gil MA, Varón C, Cardona G, Buil JA (2020). Visual acuity and defocus curves with six multifocal intraocular lenses. Int Ophthalmol.

[29] Kang KH, Song MY, Kim KY, Hwang KY, Kwon YA, Koh K (2021). Visual performance and optical quality after implantation of a new generation Monofocal intraocular Lens. Korean J Ophthalmol.

[30] Yangzes S, Kamble N, Grewal S, Grewal SPS (2020). Comparison of an aspheric monofocal intraocular lens with the new generation monofocal lens using defocus curve. Indian J Ophthalmol.

[31] Shin DY, Hwang HS, Kim HS, Kim MS, Kim EC (2021). Clinical differences between toric intraocular lens (IOL) and monofocal intraocular lens (IOL) implantation when myopia is determined as target refraction. BMC Ophthalmol.

[32] Hwang S, Lim DH, Hyun J, Kim MJ, Chung TY (2018). Myopic shift after implantation of a novel diffractive trifocal intraocular Lens in Korean eyes. Korean J Ophthalmol.

[33] Kim TI, Chung TY, Kim MJ, Lee K, Hyon JY (2020). Visual outcomes and safety after bilateral implantation of a trifocal presbyopia correcting intraocular lens in a Korean population: a prospective single-arm study. BMC Ophthalmol.

[34] Lee JH, Lee H, Lee JA, Yoo A, Kim JY, Tchah H (2020). Clinical outcomes after mix-and-match implantation of diffractive multifocal intraocular lenses with + 2.75 and + 4.00 diopter add powers. BMC Ophthalmol.

[35] Pedrotti E, Chierego C, Talli PM, Selvi F, Galzignato A, Neri E, Barosco G, Montresor A, Rodella A, Marchini G (2020). Extended depth of focus versus monofocal IOLs: objective and subjective visual outcome. J Refract Surg.

[36] Altemir-Gomez I, Millan MS, Vega F, Bartol-Puyal F, Gimenez-Calvo G, Larrosa JM, Polo V, Pablo LE, Garcia-Martin E (2020). Comparison of visual and optical quality of monofocal versus multifocal intraocular lenses. Eur J Ophthalmol.

